# How Can Promoting Skeletal Muscle Health and Exercise in Children and Adolescents Prevent Insulin Resistance and Type 2 Diabetes?

**DOI:** 10.3390/life14091198

**Published:** 2024-09-21

**Authors:** Valeria Calcaterra, Vittoria Carlotta Magenes, Alice Bianchi, Virginia Rossi, Alessandro Gatti, Luca Marin, Matteo Vandoni, Gianvincenzo Zuccotti

**Affiliations:** 1 Department of Internal Medicine and Therapeutics, University of Pavia, 27100 Pavia, Italy; 2 Pediatric Department, Buzzi Children’s Hospital, 20154 Milano, Italy; vittoria.magenes@unimi.it (V.C.M.); alice.bianchi1@unimi.it (A.B.); virginia.rossi@unimi.it (V.R.); gianvincenzo.zuccotti@unimi.it (G.Z.); 3 Laboratory of Adapted Motor Activity (LAMA), Department of Public Health, Experimental Medicine and Forensic Science, University of Pavia, 27100 Pavia, Italy; alessandro.gatti08@universitadipavia.it (A.G.); luca.marin@universitadipavia.it (L.M.); matteo.vandoni@unipv.it (M.V.); 4 Department of Biomedical and Clinical Science, University of Milano, 20157 Milano, Italy

**Keywords:** adolescents, children, exercise, health, insulin resistance, skeletal muscle, type 2 diabetes

## Abstract

Skeletal muscle secretome, through its paracrine and endocrine functions, contributes to the maintenance and regulation of overall physiological health. We conducted a narrative review on the role of skeletal muscle and exercise in maintaining glucose homeostasis, driving insulin resistance (IR), and preventing type 2 diabetes in pediatric populations, especially in the context of overweight and obesity. Myokines such as interleukin (IL)-6, IL-8, and IL-15, as well as irisin, myonectin, and myostatin, appear to play a crucial role in IR. Skeletal muscle can also become a target of obesity-induced and IR-induced inflammation. In the correlation between muscle, IR, and inflammation, the role of infiltration of the immune cells and the microvasculature may also be considered. It remains unclear which exercise approach is the best; however, combining aerobic exercise with resistance training seems to be the most effective strategy for managing IR, with high-intensity activities offering superior metabolic benefits and long-term adherence. Encouraging daily participation in enjoyable and engaging exercise is key for long-term commitment and effective glucose metabolism management. Promoting physical activity in children and adolescents must be a top priority for public health, not only in terms of individual quality of life and well-being but also for community health.

## 1. Introduction

Muscles and bones, which form the musculoskeletal system, account for roughly 55% of a healthy adult’s body weight. This system is crucial not only for enabling movement but also for supporting metabolic health. It achieves this by efficiently utilizing, distributing, and delivering nutrients and other essential substrates [1,2,3].

Physical exercise confers benefits across the entire body by reducing fat mass, enhancing insulin sensitivity, and boosting cardiopulmonary capacity, cerebral blood flow, brain oxygenation, and muscle mass and strength [4]. These effects are primarily attributed to metabolic changes triggered by physical activity, which are regulated by molecules responsible for maintaining cellular homeostasis. Specifically, exercise provides metabolic advantages and serves as a potentially effective non-pharmacological strategy against diabetes and cardiovascular diseases [5].

Research indicates that the protective benefits of physical activity against metabolic and cardiovascular disease and other chronic degenerative conditions can be partly attributed to the anti-inflammatory effects of regular exercise. The identification of myokines produced and released by muscles provides a biological basis for understanding how exercise impacts metabolism and reduces inflammation [6,7].

Interestingly, recent studies have shown that muscle exercise can promote the secretion of anti-inflammatory myokines, such as interleukin (IL)-6, IL-15, myostatin, irisin, and myonectin, able to contrast the pro-inflammatory state induced by overweight and obesity and the proper of insulin resistance (IR) [8,9,10]. 

IR plays a significant role in linking obesity to various metabolic and cardiovascular complications [11]. Pediatric patients with overweight or obesity often exhibit hyperinsulinemia and demonstrate around a 40% reduction in insulin-stimulated glucose uptake compared to children with normal weight. IR is strongly associated with an elevated risk of developing type 2 diabetes (T2D) [12,13,14]. The progression of impaired glucose tolerance in individuals with obesity is tied to the exacerbation of IR and represents a transitional phase in the natural development of T2D [5].

Regular physical exercise enhances muscle glucose uptake and increases the expression of GLUT4, a glucose transporter, in muscle cells, thereby improving insulin sensitivity and reducing the risk of developing insulin resistance and T2D [15,16]. However, it is not clear what type of exercise is most effective in reducing IR in children and adolescents [17].

We propose to present a narrative review on the role of skeletal muscle as an endocrine organ capable of influencing metabolic processes, focusing on the effects of exercise on IR and T2D in pediatrics, particularly in conditions of overweight and obesity. Promoting skeletal muscle health in children and adolescents may be useful to prevent IR and T2D. Implementing healthy lifestyle programs early on provides a more effective strategy for maintaining metabolic health and overall well-being in adulthood.

## 2. Methods

We conducted a narrative review [18], offering a non-systematic examination of the literature on the role of skeletal muscle and exercise in maintaining glucose homeostasis and driving IR in pediatric populations, especially in the context of overweight and obesity. To narrow the focus of the review, we selected the most pertinent original research articles, clinical trials, meta-analyses, and reviews published in English on this topic up to July 2024. Case reports, case series, and letters were excluded from consideration. Regarding the role of physical exercise on IR and T2D, we considered only the manuscripts that included pediatric patients (<19 years). The search terms included (alone and/or in combination) skeletal muscle, physical activity, insulin resistance, glucose metabolism, exercise, inflammation, adolescents, children, and obesity. For research purposes, we utilized PubMed and Scopus as electronic databases. 

The authors screened the abstracts of the available studies (*n* = 242). These were refined by reviewing abstracts (*n* = 173) and conducting thorough full-text assessments of pertinent studies (*n* = 162), which were critically evaluated for inclusion in the manuscript (*n* = 156). Furthermore, the reference lists of all articles were examined to identify additional relevant studies. 

## 3. Physiology of Muscle Health and the Myokines Network 

### 3.1. Skeletal Muscle as an Endocrine Organ

The idea that the contraction of muscle cells can stimulate the production of humoral factors capable of influencing metabolic processes was first introduced by Goldstein in 1961 [19]. This idea was subsequently validated, and Pedersen et al. pioneered the identification of these molecules as ‘myokines,’ as cytokines that are produced and released by muscle fibers [20]. Since then, the repertoire of recognized myokines has been steadily increasing, and the definition has broadened to encompass any protein secreted by skeletal muscle, whether it functions in an autocrine, paracrine, or endocrine manner through intercellular communication [21,22]. Nevertheless, while it is common practice to use the term “myokine” regardless of the validation of muscle fibers as the origin of the released protein, not all cytokines regulated by exercise are localized within myofibers. Satellite cells, fibroblasts, endothelial cells, and macrophages residing in muscle tissue can also contribute to the release of “myokines” [23].

Several studies on global protein profiling have provided a comprehensive description of the proteins secreted by human myotubes [23,24,25,26,27]. These analyses have revealed that many secreted myokines play a crucial paracrine role in skeletal muscle development and regeneration, extracellular matrix (ECM) organization, and angiogenesis. Additionally, other myokines have been identified to function in an endocrine capacity, primarily involved in regulating glucose and lipid metabolism, as well as neural function [23]. With regard to their impact on the nervous system, the release of these effector molecules during physical activity appears to influence mood, eating behavior, learning, and locomotor activity, also offering neuroprotection in in vivo and in vitro models [4].

On the other hand, physical inactivity fosters an imbalance among these substances, tipping the scale towards a pro-inflammatory state. This, in turn, perpetuates a vicious cycle of sarcopenia, fat accumulation—particularly visceral fat—and the development of various chronic conditions such as cardiovascular disease, T2D, cancer, dementia, and depression, a phenomenon referred to as “the disease of physical inactivity” [28].

Pedersen’s work suggests that during muscle contraction, skeletal muscles release myokines that function hormonally, exerting targeted endocrine on visceral fat [7], providing a biological basis for understanding the role of exercise on metabolism and reducing inflammation [6,7].

McPherron et al. [29] identified myostatin (MSTN) as the first myokine, which is produced and secreted by skeletal muscle. Myostatin is a highly conserved member of the Transforming Growth Factor (TGF)-β superfamily, and it is considered to have the most significant impact on muscle mass and body fat composition among known myokines, and it serves as an inhibitor of muscle mass gain [22,27,29,30]. In fact, inactivation of the myostatin gene (knockout) leads to significant skeletal muscle hypertrophy in mice, cattle, and humans [22]. Schuelke et al. [31] demonstrated that mutations in the human MSTN gene lead to reduced production of mature myostatin, resulting in increased muscle mass and a concomitant reduction in adipose tissue. Moreover, it inhibits bone healing; on the other hand, myostatin inhibitors have been shown to enhance fracture recovery [32]. Furthermore, regular exercise has been shown to reduce myostatin transcript levels in the skeletal muscle of individuals with obesity and impaired glycemic control. A decrease in myostatin mRNA levels has also been observed following acute exercise in healthy individuals. Additionally, MSTN is regulated in vivo by follistatin (FST) and decorin (DCN), which will be discussed later [33].

Several are the cytokines produced and secreted after physical exercise; an increase in cytokine concentration within the muscle interstitial fluid can be observed after 30 min of physical activity [23,34]. The type of physical activity that elicits a greater systemic cytokine response is typically associated with a higher degree of muscle damage, such as downhill running, eccentric exercise, and resistance training, as well as prolonged or high-intensity workouts [23,35].

However, in the case of IL-6, this pattern does not apply: the increase in its expression and release occurs independently of muscle damage [23]. In fact, its production and secretion appear to be regulated by carbohydrate availability and have been proposed as indicators of the muscle’s metabolic state. Low muscle glycogen content prior to exercise results in higher levels of IL-6 and interleukin-8 (IL-8) transcription after exercise. Moreover, carbohydrate ingestion before exercise attenuates the increase in these transcripts [23].

IL-6 plays a key role in both autocrine and paracrine signaling within skeletal muscle, particularly through the activation of the signal transducer and activator of the transcription 3 (STAT3) pathway, which has been observed in human satellite cells following muscle contraction [36]. In mice, IL-6 is crucial for hypertrophic muscle growth and myogenesis [37].

The endocrine effects of exercise-induced IL-6 release are central to the concept of health-promoting myokines. IL-6 enhances insulin-stimulated glucose uptake and oxidation, stimulates lipolysis and fat oxidation, promotes pancreatic β-cell expansion, and improves insulin secretion and glycemic control by stimulating glucagon-like peptide (GLP)-1 release from intestinal L-cells and pancreatic α-cells [4,38,39,40]. In fact, within the skeletal muscle, IL-6 activates AMP-activated protein kinase (AMPK) and/or phosphatidylinositol 3 (PI3K)-kinase pathways to enhance glucose uptake and fat oxidation. Additionally, IL-6 is released into circulation, where it reaches the liver to stimulate glucose production during exercise and the adipose tissue to promote lipolysis [28]. Additionally, IL-6 supports alternative macrophage activation, which is protective against obesity-induced tissue inflammation and insulin resistance [41].

Recent studies also suggest that exercise can reduce tumor size and growth in mice by mobilizing IL-6-dependent natural killer cells [42]. Overall, IL-6 is associated with several beneficial effects of exercise, including improved glycemic control, fat loss, tumor suppression, and muscle mass maintenance.

IL-6 has commonly been considered a pro-inflammatory cytokine, mainly because of its role when released by monocytes and macrophages in reaction to infection. Nevertheless, when IL-6 is secreted by skeletal muscle, it happens without the presence of other inflammatory factors like IL-10 and tumor necrosis factor (TNF)-α. This suggests that the cytokine response induced by physical exercise does not align with typical inflammation pathways [28]. Exercise increases circulating levels of anti-inflammatory cytokines, such as IL-1ra, IL-10, and soluble TNF receptors, which act as natural inhibitors of TNF-α. During exercise, IL-6 itself may exert anti-inflammatory effects, as evidence suggests that this myokine can suppress the synthesis of IL-1 and TNF-α and stimulate the production of IL-1ra and IL-10 [28].

Not only are IL-6 and myostatin regulated by physical activity and involved in post-exercise metabolic regulation, but several other myokines also play critical roles in reducing subcutaneous and visceral fat and enhancing substrate oxidation capacity, both of which are essential for insulin sensitivity [4].

IL-15, a member of the IL-2 superfamily, is another chemokine produced by skeletal muscle [4,28]. IL-15 signaling appears to be involved in the regulation of muscle fiber composition and contractility, acting as an anabolic factor that stimulates muscle growth. Additionally, IL-15 plays a role in lipid metabolism regulation [4,28]. It has been shown to reduce lipid accumulation in preadipocytes and decrease the mass of white adipose tissue. As a result, a negative relationship has been observed between plasma IL-15 levels and total body fat mass, trunk fat mass, and the percentage of body fat in humans, and overexpression of IL-15 in skeletal muscle has been shown to reduce visceral fat in murine models [4,28]. 

Additional exercise-regulated myokines promote the proliferation of primary human skeletal muscle cells, such as decorin, leukemia inhibitory factor (LIF), and chitinase-3-like protein 1 (CHI3L1) [43,44,45].

Decorin, a small leucine-rich proteoglycan, is synthesized and secreted by human myotubes in response to muscle contraction, with its levels increasing following physical exercise. It has been demonstrated to inhibit myostatin activity in both fibroblasts and myoblasts and also influences the expression of follistatin, another inhibitor of myostatin [33,43]. In fact, mouse studies have shown that overexpression of decorin in skeletal muscle in vivo enhances the expression of the pro-myogenic factor Mighty, which is downregulated by myostatin. Additionally, Myogenic Differentiation 1 (Myod1) and follistatin expression are elevated in response to decorin overexpression [33,46]. In summary, decorin, which is secreted by myotubes in response to exercise, plays a role in regulating muscle hypertrophy and may contribute to the remodeling of skeletal muscle during exercise.

The myokine LIF is part of the IL-6 cytokine superfamily, which comprises structurally and functionally similar proteins known as neuropoietins (or gp130 cytokines) [47]. It is a contraction-induced myokine, acting in an autocrine and/or paracrine fashion [22]. LIF plays a diverse role in biological processes, including promoting platelet production, the proliferation of hematopoietic cells, bone formation, neural survival and development, and the acute-phase response in liver cells [48]. Moreover, it supports satellite cell proliferation [22]. The expression of LIF mRNA is triggered in human skeletal muscle after resistance exercise, and the LIF protein is released by electrically stimulated human myotubes in culture [44]. LIF enhances the proliferation of human myoblasts and triggers the expression of jun-B and c-Myc in human myotubes. In contrast, knocking down the LIF receptor using siRNA led to decreased proliferation [22]. 

CHI3L1 (chitinase-3-like protein 1) is a glycoprotein composed of 383 amino acids with a molecular mass of 40 kDa [45,49,50]. While it is well-known as an acute-phase protein that increases during inflammatory diseases, particularly sepsis, CHI3L1 also plays several other roles [45,50]. Notably, it is produced and secreted by skeletal muscle in response to acute physical exercise. The CHI3L1/PAR-2 signaling pathway, in turn, stimulates myocyte proliferation, which is crucial for skeletal muscle remodeling in response to training [45,50]. Additionally, CHI3L1 has been linked to enhanced glucose uptake in skeletal muscles through an AMP-activated protein kinase (AMPK)-dependent mechanism and an increase in intracellular calcium levels via PAR2 [49]. Furthermore, CHI3L1 has been found to influence glucose uptake through the PI3K/AKT pathway [49].

Angiopoietin-like protein 4 (ANGPTL4), part of the angiogenin-like protein family, is expressed across a diverse array of tissues, including the liver, adipose tissue, skeletal muscle, placenta, small intestine, brain, thyroid, kidney, spleen, pituitary gland, hypothalamus, and heart. It is upregulated in response to various physiological conditions, including fasting and physical exercise. Following acute resistance and strength training, ANGPTL4 mRNA levels rise in both serum and skeletal muscle. In non-exercising muscle, the increase in ANGPTL4 is mediated by elevated plasma free fatty acids (FFAs) through PPAR activation, likely to prevent fat overload and ensure the supply of fatty acids to active skeletal muscles [51,52,53]. Given that ANGPTL4 regulates lipid metabolism by inhibiting lipoprotein lipase activity and promoting lipolysis in white adipose tissue, it may contribute to insulin resistance by regulating its expression through PPARs (PPARα, PPARδ/β, PPARγ) [54,55]. A study by van der Kolk et al. [56] examined the impact of a mixed meal rich in saturated fatty acids (SFA) on plasma ANGPTL4 levels and its relationship with muscle LPL activity in vivo, aiming to understand the effects of dietary fat quality and the release of ANGPTL4 from skeletal muscle. The study found that ANGPTL4 is secreted from human forearm muscle after a high-SFA meal in postprandial conditions. However, plasma ANGPTL4 levels were not linked to skeletal muscle LPL activity in vivo following the SFA-rich meal. While dietary fat quality does affect plasma ANGPTL4, its impact on short-term lipid metabolism in skeletal muscle remains to be determined [56]. Additionally, ANGPTL4 has recently been implicated in mice as a mediator of pancreatic β-cell hyperplasia [4,53].

Apelin is a peptide produced by skeletal muscles, with its production triggered by muscle contractions [57]. It is a ligand for the G protein-coupled receptor APJ, and it has been shown in rodents to enhance glucose uptake and mitochondrial oxidative capacity in skeletal muscle [58,59,60]. Furthermore, its levels decline with age in both humans and animals, and mice lacking apelin or its receptor (APLNR) experience significant muscle function deterioration as they age [57]. Restoring apelin activity in these aging mice improves muscle function by enhancing energy production, reducing inflammation, and supporting muscle stem cell (MuSC) regeneration. These findings make apelin a potential biomarker for early muscle deterioration and a target for therapies to combat age-related muscle weakness [57]. In related research, Le Moal et al. [61] identified a specific form of apelin (AP-13) that stimulates skeletal muscle endothelial cells (ECs), improving muscle regeneration and strength in mouse models of muscular dystrophy by creating a supportive environment for MuSCs. This research suggests that targeting apelin signaling, particularly through AP-13, may offer a promising strategy for treating muscle stem cell dysfunction and enhancing muscle repair.

Irisin is a recently identified polypeptide hormone, discovered also in human plasma via mass spectrometry [62], and induced by physical exercise and secreted by muscle. It is produced through the proteolytic processing of FNDC5 [63], whose expression in muscle is, in turn, upregulated by PGC-1α. Irisin was discovered as a myokine that promotes the transformation of white adipose tissue into a brown fat-like phenotype [22]. In mice, the injection of adenoviral particles expressing FNDC5 led to a three- to four-fold increase in irisin levels, resulting in the induction of a brown fat-like cell development program in white adipose tissue and a concomitant increase in energy expenditure [22,63]. Baseline plasma levels of irisin were found to increase in response to 10 weeks of regular physical exercise in humans, suggesting a role for irisin in exercise adaptation. Additionally, irisin has been shown to exert antifibrotic effects on the heart, liver, pancreas, and muscle. Specifically, its antifibrotic effect on muscle was demonstrated in a recent study by Wu et al. [64]. In their study examining the impact of irisin on D-galactose (D-gal)-induced skeletal muscle fibroblasts, it was found that irisin or FNDC5 overexpression effectively inhibited or reduced the effects of D-galactose, such as cellular senescence, fibrosis, and redox imbalance. This protective effect was achieved through modulation of the phosphatidylinositol 3-kinase (PI3K)/protein kinase B (Akt) signaling pathway [64].

Myokines produced and secreted in response to physical activity have also been linked to direct cardiovascular benefits, including increased cardiac output and overall blood volume. Notable among these are vascular endothelial growth factor (VEGF), as well as IL-8, which promotes endothelial cell proliferation and capillary tube formation, and CYR61 (CCN1) and CTGF (CCN2), which are associated with the extracellular matrix (ECM) protein group [4]. 

During physical exercise, skeletal muscle fibers synthesize and secrete VEGF within vesicles. VEGF is a primary angiogenic factor that plays a crucial role in the development of blood vessels, including the capillaries within skeletal muscle tissue. During muscle contraction, VEGF concentration increases within the muscle interstitium, thereby stimulating angiogenic processes [65]. Moreover, VEGF not only enhances blood vessel growth but also plays a significant role in tissue regeneration [66]. On the other hand, CYR61 (CCN1) and CTGF (CCN2) increase in skeletal muscle following exercise, especially following mechanical loading [67]. They are involved in skeletal muscle remodeling after exercise, as they regulate the expression of genes involved in angiogenesis and ECM remodeling.

The myokine brain-derived neurotrophic factor (BDNF), which increases following physical activity, plays a critical role in neural processes by regulating the growth, survival, and maintenance of neurons [28]. This, in turn, influences information processing, learning, and memory. Studies on individuals with Alzheimer’s disease have shown low plasma concentrations of BDNF, and post-mortem analysis of hippocampal samples has revealed reduced BDNF expression [28,68]. Additionally, low blood levels of BDNF have been associated with major depressive disorder, acute coronary syndromes, and type 2 diabetes mellitus [28]. BDNF also influences energy metabolism and adipose tissue by enhancing fat oxidation through an AMPK-dependent pathway in skeletal muscles, leading to a reduction in adipose tissue mass [69].

In Table 1, different myokines and their roles are resumed. 

### 3.2. Skeletal Muscle and Glucose Metabolism

Skeletal muscle uses three main types of substrates for its energy metabolism: glycogen, glucose, and free fatty acids [70]. The specific energy sources used by muscles depend on several factors, including physical conditioning, diet, type, intensity, and duration of exercise [71]. At rest, in fact, the main energy sources are fatty acids, whereas, during exercise, the energy metabolism of skeletal muscle depends on both glucose and free fatty acids. 

According to the intensity of exercise, the major energy substrate is glucose during high-intensity isometric exercise, glucose and glycogen in the case of high-intensity submaximal exercise, and both glucose and free fatty acids in the case of low-intensity submaximal exercise. According to the duration of exercise, instead, in the first hour of mild activity, the primary sources of energy are glucose, glycogen, and free fatty acids, whereas in the following hours, the uptake of free fatty acids increases gradually. Therefore, lipid metabolism plays a predominant role at rest, whereas glucose metabolism is crucial for energy generation during exercise [72]. 

Skeletal muscle accounts for more than 80% of glucose absorption in the post-meal state [73], playing a vital role in regulating blood glucose levels. It absorbs glucose from the extracellular fluid into the cell via specific sugar transport proteins located on the cell membrane [8,74], known as GLUTs (facilitative glucose transporters) and SGLTs (sodium-dependent glucose co-transporters).

There are three main isoforms of GLUTs in skeletal muscle: the most important one is GLUT4, followed by GLUT1 and GLUT3, which are expressed only in fetal and neonatal muscle [8,75]. Other sugar transporter proteins expressed in skeletal muscle include GLUT5, GLUT6, GLUT8, GLUT10, GLUT11, GLUT12, SGLT1, SGLT2, SGLT3, and SGLT4 [76]. In contrast to GLUT1, which is found on the surface of muscle cells, GLUT4 is an intracellular protein that requires stimulation, such as from insulin or physical exercise, to move to the cell surface and assist in glucose absorption [77]. In patients with type 2 diabetes or insulin resistance, the ability of insulin to stimulate skeletal muscle glucose transport is altered [78,79] due to an impaired GLUT4 translocation to the muscle cell surface [80]. Exercise, on the contrary, can stimulate GLUT4 translocation and glucose transport, both in healthy and type 2 diabetes individuals [81]. 

**Figure 1:**
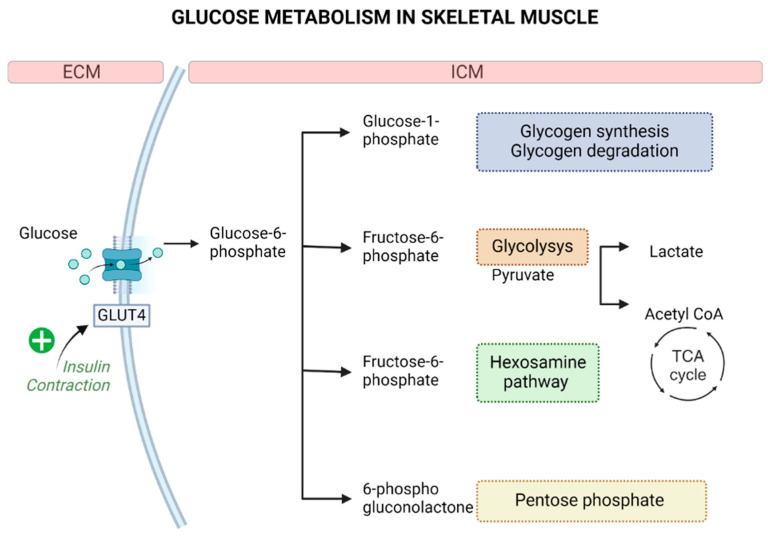
Glucose metabolism in skeletal muscle. Legend: Created by Biorender®. ECM: extracellular matrix; ICM: intracellular matrix.

Once transported into skeletal muscle cells, glucose is phosphorylated by hexokinase to glucose-6-phosphate, and then it can go through four different intracellular pathways, as described in Figure 1.

Glycogen, the stored form of glucose in skeletal muscle, acts as a glucose buffer. At rest, muscle glycogen levels are regulated by the balance between glycogen synthesis and degradation. However, exercise leads to an increase in glycogen synthesis [82]. During this process, glucose-6-phosphate is first converted to glucose-1-phosphate by the enzyme phosphoglucomutase and then to uridine diphosphate glucose (UDP-glucose) by glycogen synthase (GS), forming the multi-branched glucose polymers characteristic of glycogen particles. When cellular energy demands rise, glycogen is broken down into glucose-1-phosphate by glycogen phosphorylase (GP) and subsequently metabolized through glycolysis [76].

Glucose flux through glycolysis plays a fundamental role in generating adenosine triphosphate (ATP), which is the source of energy for skeletal muscle contractile function. Glucose-6-phosphate is converted to fructose-6-phosphate by the enzyme phosphoglucose isomerase and then to fructose-1,6-bisphosphate by the enzyme phosphofructokinase (PFK). After seven other sequential reactions, fructose-1,6-bisphosphate is finally converted to pyruvate. Pyruvate can be reduced to lactate or, on the other hand, can be oxidated to acetyl-CoA by the enzyme pyruvate dehydrogenase (PDH) and further metabolized via the tricarboxylic acid cycle (TCA) and mitochondrial electron transport chain [76]. The complete oxidation of glucose generates 36 molecules of ATP.

The hexosamine pathway is a glucose-utilizing pathway that starts when fructose-6-phosphate is converted to glucosamine-6-phosphate by the enzyme glutamine fructose-6-phosphate transaminase 1 (GFPT1). This biosynthesis pathway determines the production of uridine diphosphate-*N*-acetyl glucosamine (UDP-GlcNAc), which is a key metabolite for *N*- or *O*-linked glycosylation, which modulates protein expression and activity [83].

The last glucose-utilizing pathway in skeletal muscle is the pentose phosphate pathway. Glucose-6-phosphate is converted to 6-phosphogluconolactone by the enzyme glucose-6-phosphate dehydrogenase (G6PD). Then, it is metabolized through a series of reactions in order to generate important metabolites, such as nicotinamide adenine dinucleotide phosphate (NADPH), which is crucial for reductive biosynthesis reactions such as lipogenesis; ribose 5-phosphate, used for nucleotide synthesis; erythrose-4-phosphate, used for aromatic amino acid synthesis; and ribulose 5-phosphate, often measured to check pentose phosphate pathway activity [84]. At rest, the activity of the pentose phosphate pathway is low in skeletal muscle [85] since it is a differentiated cell-type tissue and does not have high biosynthetic demands. On the contrary, studies showed that the activity of the pentose phosphate pathway increases after damage to provide substrates for muscle repair processes [76].

### 3.3. Key Myokines in the Regulation of Glucose Metabolism

Skeletal muscle, being a key player in the regulation of glucose metabolism, is also considered the primary driver of whole-body IR. If the main metabolic defect occurs at the level of skeletal muscle, restoring glucose homeostasis by addressing IR in the muscle is fundamental and may be sufficient [73].

IR occurs due to the desensitization of muscle to the action of insulin (to elicit glucose uptake), and it leads to unproperly elevated blood glucose levels [86]. Interestingly, IR in skeletal muscle can appear decades before the onset of pancreatic β-cell failure and symptomatic T2D [8,73,87]. 

IR negatively affects the glucose uptake at the skeletal muscle levels both in terms of timing and in terms of quantity [8,73]. Indeed, in physiological conditions, postprandial glucose uptake—upon insulin release—increases into muscle linearly with time [8,73,88]. Instead, in a condition of IR, insulin action and glucose uptake are delayed, and this leads to a diminished overall glucose uptake by the skeletal muscle [8,73,88]. This has been shown by hyperinsulinemic-euglycemic clamping studies in patients affected by T2D and controls [8,89].

Among the myokines produced by the muscle IL-6, IL-15, and interleukin-8 (IL-8), [1,15,17], as well as irisin, myonectin, and myostatin [1,15,17] appear to play a crucial role in IR. No other exercise-induced molecules were more extensively evaluated, in this context, in the pediatric field.

Specifically, IL-6 is one of the best-known pro-inflammatory cytokines released by skeletal muscle, and it was shown to contribute to the onset of IR [90,91]. Recently, this molecule was shown to also have an anti-inflammatory effect when released in response to exercise [92]. The different effects are due to the activation of different signaling pathways. Specifically, IL-6 acts as a pro-inflammatory molecule through the Nuclear Factor kappa (NFκB) pathway, but it has an anti-inflammatory potential when released through a calcium-dependent Mitogen-activated Protein Kinase (MAPK) signal [92,93]. Hojman et al. investigated the mechanism involved in the exercise-mediated surge in IL-6 and the related lactate production, evaluating muscle activity upon interval-based cycling in healthy young men, swimming exercise in mice, and electrical stimulation in human muscle cells [94]. The authors discovered that IL-6 levels rise with both the intensity and duration of exercise. Additionally, the release of IL-6 during exercise is influenced by protease activity and lactate production [94].

Similarly, IL-15 is a myokine known to be regulated by exercise and muscle contraction [8,95]. It has been associated with obesity and metabolic syndrome [8,95,96]. This molecule is thought to mediate the benefits of exercise, but the precise effect of muscle activity on IL-15 stimulation varies from study to study [97,98,99,100]. Recently, Khalafi et al. performed a systematic review and meta-analysis to investigate whether exercise (acute and chronic) stimulates an increase in circulating IL-15 concentrations in humans [101]. The authors evaluated a total of 27 studies involving 1310 participants and showed that acute exercise increases circulating IL-15 concentrations immediately and one hour after [101]. Instead, chronic exercise does not have a significant effect on IL-15 concentrations [101]. Thus, the results confirm that acute exercise, increasing IL-15 concentrations, has a potential role in improving metabolism in adults [101]. The ongoing debate about IL-15’s effects highlights the need for further research to clarify the precise role of this myokine [8,95].

Unfortunately, studies evaluating exercise-induced interleukins and their role in me-tabolism in children and adolescents are scarce. Thus, even if the results on adults are promising, further works are needed to better understand the role of these molecules in glucidic metabolism in these patients and how these molecules specifically act.

Myostatin was the first myokine identified. It is part of the TGF-β family of proteins [8,9,102], and it is expressed both in skeletal muscle and in adipose tissue [103]. Myostatin negatively regulates muscular mass. Indeed, its ablation was shown to be correlated to muscle hypertrophy [104,105]. Moreover, growth differentiation factor 8 negatively regulates muscular mass [105,106]. Indeed, its ablation was shown to be correlated to muscle hypertrophy [104,105]. Upon exercise, myostatin decreases, and its expression has been correlated to a decrease in plasma levels of glucose, insulin, and IL-6 and a reduction in IR (calculated with the Homeostatic Model Assessment for Insulin Resistance Index, HOMA-IR) [106,107,108]. Further studies are anyway needed to better understand its correlation with glucose metabolism and eventually consider this molecule as a candidate therapeutic target to combat obesity by promoting muscle growth [8].

Irisin expression, upon muscular exercise, is thought to be involved in the beginning of white adipose tissue, causing white adipose tissue to partially transform into the brown adipose tissue phenotype [62,63,109,110]. As brown tissue is associated with increased thermogenesis, this phenomenon can lead to improvements in terms of obesity and glucose homeostasis [63]. Interestingly, Jedrychowski et al. identified and quantitated human irisin in plasma (using mass spectrometry) in individuals undergoing aerobic interval training [62]. The authors showed that circulating irisin levels significantly increased with exercise and that mass spectrometry can be a possible tool to measure it [62].

Reinehr et al. evaluated 40 children with obesity and 20 normal-weight children (of similar age and gender) after a 1-year outpatient intervention program based on exercise, behavior, and nutrition therapy in order to study the relationships between irisin, puberty and IR after weight loss [110]. The authors evidenced that irisin levels were the highest in children with obesity and with impaired glucose tolerance and the lowest in normal weight children. In addition, their longitudinal analyses showed that changes of irisin were associated with entry into puberty and amelioration of glucidic metabolism (change of fasting glucose, and 2-hour glucose in an oral glucose tolerance test), but not with change of BMI [110].

However, there is controversy regarding the increase in serum irisin following muscular activity, both due to the difficulty in detecting irisin in the blood and the scarcity of studies on this subject, particularly in a pediatric context.

Myonectin is a nutrient-responsive myokine released by muscle contraction that mimics insulin’s ability to promote fatty acid cellular uptake response to exercise [109,110,111]. In addition, myonectin can also increase the translocation of the GLUT4 glucose transporter and promote glucose uptake [112]. Lastly, this molecule has been associated with mitochondrial deoxyribonucleic acid (mtDNA) density; specifically, IR, leading to decreased mtDNA levels, can upregulate myonectin [112]. Even in this case, further studies are needed to clearly understand the role of this molecule in this context [8].

Finally, other myokines shown to have a role in glucose metabolism and muscle include BDNF and Decorin [8,10,112]. Moreover, new myokines that respond to exercise (called “exerkines”) have been identified [113]; their effect on IR must be clarified, especially in the pediatric field, before they can be lev-eraged as potential therapeutic targets.

**Figure 2:**
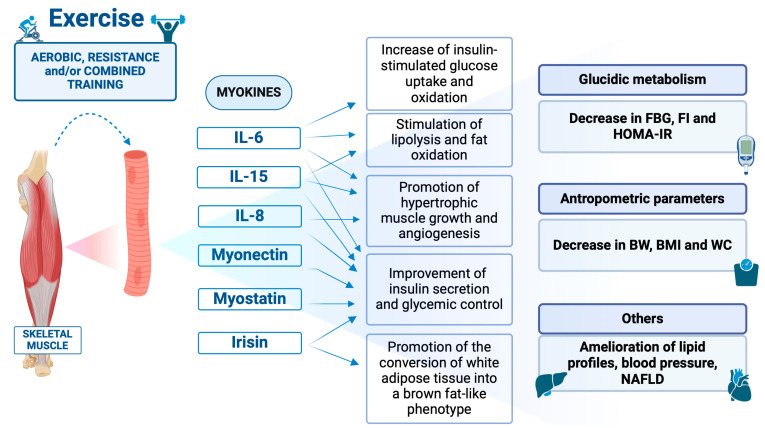
Key myokines involved in muscle contraction and influencing glucose metabolism. Created by BioRender®. FBG = fasting blood glucose; FI = fasting insulin; HOMA-IR = Homeostatic Model Assessment for Insulin Resistance index; BW = body weight; BMI = body mass index; WC = waist circumference; NAFLD = Non-alcoholic fatty liver disease.

In Figure 2, the properties of the key myokines involved in muscle contraction and in-fluencing glucose metabolism are schematized.

IR is strictly correlated to weight gain, overnutrition, and obesity; it has been shown that when lean and non-diabetic individuals were given a regimen of overnutrition, they developed IR [114]. The link between IR and obesity may be partially explained by the condition of chronic inflammation proper of both conditions [8,115]. Indeed, adipose tissue releases specific pro-inflammatory bioactive molecules (called adipokines) that have been shown to be dysregulated in conditions of obesity, IR, and diabetes [116,117,118]. 

Myokines are molecules that facilitate communication between skeletal muscle and other organs such as bone, brain, and adipose tissue [8,9]. Moreover, skeletal muscle itself can become a target for inflammation induced by obesity and IR [8]. Both obesity and IR have been found to stimulate the release of specific cytokine hormones from tissues, known as adipomyokines [103]. Unlike adipokines, which are influenced by factors like body fat, muscle myokines are primarily regulated by exercise and muscle contractions [8,9]. The levels of these circulating factors can vary in response to conditions such as obesity, inflammation, and IR [8,9].

Additionally, in conditions of obesity, IR and/or T2D skeletal muscle becomes infiltrated by immune cells (mainly M1-like macrophages and T cells) [119,120]. Moreover, its adipose tissue depots can expand for up to 10% of the total muscle mass [119]. These depots—intramuscular or subcutaneous—are thought to be the main contributors of pro-inflammatory immune cells in the skeletal muscle [119], and they have been correlated to glucose intolerance and IR [1,19,20,21]. 

Examining the correlation between muscle, IR, and inflammation, it is worth considering also the role of microvasculature [121,122]. Indeed, evidence confirms that muscle microvasculature is a fundamental site of action of insulin, and it regulates insulin delivery and action on myocytes, thus affecting insulin-mediated glucose disposal [121,122]. IR at the microvascular level due to inflammation is an early event in the development of metabolic IR and eventually T2D; thus, research should nowadays focus on this theme in order to find new potential therapeutical targets [121].

In this context, the role of thiazolidinediones (TZDs) has to also be considered. TZDs, such as pioglitazone and rosiglitazone, help to improve insulin sensitivity by targeting peroxisome proliferator-activated receptor gamma (PPARγ) receptors, which are expressed in adipose tissue but also influence muscle metabolism indirectly [123,124]. Indeed, in skeletal muscle, TZDs enhance insulin-mediated glucose uptake by improving the action of insulin signaling pathways, thus reducing IR [123,124]. 

In women and adolescents with polycystic ovary syndrome (PCOS), IR in skeletal muscle exacerbates hyperinsulinemia, which, in turn, stimulates excess androgen production by the ovaries, worsening reproductive symptoms [125,126]. The use of TZDs in these patients was shown to improve metabolic outcomes by enhancing insulin sensitivity in skeletal muscle [127,128,129]. In young patients affected by PCOS, metformin remains the most effective and cost-efficient therapy to regulate insulin sensitivity in the condition of overweight and/or obesity [129,130]. While there is evidence supporting TZD efficacy in young women and adolescents with PCOS, further studies are necessary to confirm the results obtained and evaluate long-term effects. 

Finally, it is noteworthy that the relationship between skeletal muscle and IR appears to be partially influenced by gender. Recently, Liu et al. explored the gender-specific associations between predicted skeletal muscle mass index (pSMI) and the incidence of type 2 diabetes in a longitudinal cohort study of Chinese adults. The study, which utilized the WATCH (West China Adult Health Cohort) database, included Chinese men and women without diabetes at the study’s start. The researchers calculated pSMI, measured blood glucose levels, and collected self-reported medical histories to identify new cases of diabetes [131]. Then, they concluded that in females, a larger muscular mass is associated with a lower risk of T2D; instead, for males, this association is significant only among those with diminished muscle mass [131]. Moreover, Ciarambino et al. conducted a systematic literature search to identify studies evaluating the association between gender and IR [132]. The authors found that IR is generally more prevalent in men compared to premenopausal women. Instead, after menopause, the incidence of IR in women increases and becomes more comparable to that of men [132]. This difference seems to depend both on body fat distribution and on sexual hormones. Indeed, men tend to accumulate more visceral fat, which is strongly associated with IR and metabolic disturbances [132]. Instead, estrogen, the primary female sex hormone, appears to have protective effects on insulin sensitivity. Indeed, women tend to have better insulin sensitivity during their reproductive years due to the presence of estrogen. However, after menopause, estrogen levels decline as insulin sensitivity [132]. Unfortunately, the literature concerning sex differences in the association between skeletal, muscular mass, and glucidic metabolism is scarce in the pediatric field, and further research should be conducted to drive conclusions on this issue.

## 4. Effects of Exercise Training on Insulin Resistance and Type 2 Diabetes in Pediatrics

Exercise training is considered an important strategy to cope with IR and related metabolic unbalances [17], and this is of fundamental importance as impairment in insulin signaling is correlated to the future development of severe conditions such as T2D and metabolic syndrome, both in adults and in children or adolescents, primarily in the condition of overweight and obesity [133,134]. Exercise was shown to have favorable effects on metabolic indices (such as blood glucose and insulin levels) related to overweight and obesity acting at various levels. Overweight and excessive fat tissue is associated with IR and exercise; decreasing body weight and adipose tissue leads to a decrease in IR [135].

Specifically, with aerobic exercise, alterations in both metabolic and non-metabolic processes contribute to improvements in IR and associated indices [17,136]. Specifically, muscle contracting leads to the translocation of the glucose transporter 4 (GLUT4) to the plasma membrane and increases the density of this transporter in order to increase glucose uptake from the blood into the muscle [137,138,139,140]. The amount of glucose uptake by the skeletal muscle is determined by the activity duration and intensity: increased exercise intensity and time increase glucose uptake [8,137,138]. In addition, muscular activity has been associated with an increase in insulin receptor substrate 1 (IRS1) phosphorylation, leading to improvement of insulin signal transduction; an improvement of beta-cell and pancreatic islets function, maintaining beta cell mass and preventing apoptosis in islets; an induction of angiogenesis, leading to higher glucose uptake by myocytes; a reduction in the oxidative stress-induced insulin resistance and consumption of free fatty acids as metabolic substrate, reducing adipokine generation and consequent inflammation [17,140]. Muscular exercise may also modulate mitochondrial activity, beneficial to glucose homeostasis, and induce changes in the transcription factors involved in insulin signal transduction, which, in turn, increase insulin sensitivity [140,141,142].

IR is a strong determinant of chronic diseases in adulthood, and childhood and adolescence are vulnerable times for the development of IR and its consequences; therapeutical strategies such as intensive lifestyle modification (including improved dietary intake and muscular exercises) undertaken during or prior to puberty may have great positive effects on health outcomes, both in terms of glucose homeostasis (preventing IR and T2D) and in terms of weight control (reducing overweight and preventing obesity development) [143].

Reasonable fears related to the potential for disordered eating and eating disorders upon strict dietary modification in this population led to a larger emphasis on exercise training with respect to nutritional interventions during childhood and adolescence [144]. Indeed, physical activity has become a fundamental of obesity-induced IR treatment [145].

In general, the exercise training types evaluated in children and adolescents can be subdivided into three major categories: aerobic training (AT), resistance training (RT), and combined training (AT + RT), as shown in Table 2. 

Kazeminasab et al. performed a meta-analysis to determine the effects of exercise training (AT, RT, RT+RT) on IR and body weight in children and adolescents with overweight or obesity [17]. The authors reviewed 35 studies involving 1,550 children and adolescents aged 9 to 18 years with overweight and obesity, analyzing the effects of exercise training on fasting glucose, fasting insulin, HOMA-IR, and body weight [17]. The interventions lasted between 4 and 24 weeks, with most studies focusing on a 12-week duration. Training sessions were typically held 2 to 5 times per week, with 3 sessions per week being the most common. Each session lasted between 30 and 60 min [17]. The meta-analysis concluded that exercise is an effective strategy to decrease fasting glucose, fasting insulin, HOMA-IR, and BW in children and adolescents who are overweight or obese, and it could be considered an important strategy to control IR [17]. Moreover, the study evidenced that exercise training was effective regardless of the length of the intervention (> 8 and ≤8 weeks), biological sex (female/male), BMI percentiles (overweight/obesity), and health status (with and without other diagnoses). It is worth underscoring that the outcomes evidence were obtained with no specific caloric restriction and/or adherence to a dietary plan; this is important as adherence to a restrictive diet may be challenging for this population [17]. 

Interestingly, subgroup analyses by sex indicated a significant reduction in fasting glucose, fasting insulin, and HOMA-IR for females and males but not for females and males combined, compared with a control group [17]. 

Analyzing the type of exercise performed, the authors evidenced that significant reductions in fasting glucose, fasting insulin, and HOMA-IR were obtained both for AT and for CT, but not for RT alone, compared with controls [17]. Exercise training in children with overweight and obesity was also shown to effectively reduce body weight in these patients [17,146].

The results confirmed the data previously obtained by another systematic review and meta-analysis performed by Marson et al., who assessed the associations of aerobic, resistance, and combined exercise with changes in IR, fasting glucose, and fasting insulin in children and adolescents with overweight and obesity. Indeed, the authors showed that physical training, in general, was associated with reductions in fasting insulin levels and HOMA-IR and concluded that exercise training, especially aerobic, leading to a reduction in fasting insulin levels and HOMA-IR, may prevent metabolic syndrome and type 2 diabetes in children and adolescents with obesity and overweight [14]. Moreover, the reduction in fasting glucose and insulin upon exercise was relevant regardless of the length of the interventions. Indeed, significant results were obtained both in the case of long-term interventions (≥8 weeks) and short-term ones (<8 weeks) [17]. Differently from insulin and glucose blood levels, the reduction in body weight was significant for intervention durations of more than 8 weeks but not for durations of less than 8 weeks, and subgroup analyses by sex evidenced a significant reduction in body weight for males but not for females, compared with a control group [17].

Among aerobic training, Kim et al. included jumping rope, which was shown to decrease fasting insulin, fasting blood glucose, and HOMA-IR [147]. Moreover, this aerobic training type was also associated with a decrease in body weight and blood pressure levels [147]. Similarly, Murphy et al. evaluated dancing (in the form of exergaming) and reported beneficial effects in terms of blood pressure levels and lipid profile [148]. Vasconcellos et al. studied the effects of 12-week soccer training (60 min sessions performed 3 times/week) in terms of biochemical, cardiovascular, and fitness health markers in obese people with obesity and evidenced significant reductions in terms of BMI, waist circumference, and blood pressure levels [149]. Moreover, a better lipid blood profile (reduction in total cholesterol and triglycerides) and a decrease in insulin resistance (evaluated with HOMA-IR) were demonstrated [149]. Among resistance training, Seo et al. evaluated the effect of an 8-week yoga-asana training on body composition, lipid profile, and IR in adolescent boys with obesity [150]. The authors showed that the proposed training was effective in weight loss, improvement of body composition, and amelioration of lipid profile and IR, suggesting that yoga may be effective in controlling some metabolic syndrome factors in adolescents with obesity [150].

It remains not fully elucidated whether combined AT and RT may have greater improvements with respect to aerobic exercise alone [17] and whether high-intensity training intervention (HIIT) has more beneficial effects compared with moderate ones [17]. Precisely, HIIT is defined as an alternation of short bursts of intense physical activity, performed with a “near maximal” effort, with brief periods of rest or lower-intensity exercise [151]. Cao et al. showed not significant differences in terms of blood glucose, HOMA-IR, and body weight when comparing HIIT with moderate-intensity ones in children and adolescents [152], but HIIT intervention was shown to improve cardiorespiratory fitness more significantly than moderate-intensity training by Liu et al. [153]. 

Recently, Garcìa-Hermoso et al. performed a systematic review and network meta-analysis evaluating exercise training types in children and adolescents with a mean age of 13.5 years with excess weight in order to compare training modalities and their association with changes in IR markers [154]. Their study included works with a total of 3051 children and adolescents, 50.4% girls and 49.6% boys, and the authors confirmed that exercise was associated with reductions in FI and HOMA-IR and that a combination of HIIT and resistance training was the most effective training modalities to ameliorate IR markers [154]. Notably, the authors found a nonlinear association between exercise dose and IR markers (IR and HOMA-IR), with a minimal dosage requirement of around 900–1200 MET-min/week, which can be considered equivalent to two to three sessions per week of 1 h of moderate/vigorous training [154]. Importantly, the review also suggests that even a modest increase in weekly exercise can improve glucidic metabolism [154]. 

**Table 2:** Studies evaluating the metabolic effects of exercise (AT, RT, and combined) in overweight and/or children and adolescents with obesity.

References	Exercise Type	Age (yrs)	Duration (Weeks)	Diet	Results		
					Glucidic Metabolism	Anthropometric Parameters:	Others
Aerobic training							
Sun et al., 2011 [155]	Aerobic	Exe (F): 13.80 ± 0.60 Exe (M): 13.38 ± 0.40 Con: 13.60 ± 0.70	10	Habitual diet	No significant effect on FI and HOMA-IR	Decrease in BW	na
Kelly et al., 2004 [156]	Aerobic	Exe: 10.90 ± 1.99 Con: 11.00 ± 2.24	8	Habitual diet	Decrease in FBG and FI	Decrease in BW	Amelioration of lipid profiles
Liu et al., 2018 [157]	Aerobic	Exe: 14.60 ± 0.70 Con: 14.70 ± 0.80	4	1400–1600 kcal/day	Decrease in FBG, FI, and HOMA-IR	Decrease in BW	na
Karacabey et al., 2009 [158]	Aerobic	Exe: 11.80 ± 0.50 Con: 11.20 ± 0.80	12	Specific diet program	Decrease in FI	Decrease in BW	Decrease in cortisol levels and amelioration of lipid profile
McCormack et al., 2014 [159]	Aerobic	Exe:13.80 ± 2.20 Con:12.10 ± 1.20	8	Habitual diet	Decrease in HOMA-IR	Decrease in BW and WC	Amelioration in body composition
Kim et al., 2011 [160]	Aerobic	Exe: 17.63 ± 0.49 Con na	12	Habitual diet	Decrease in FBG, FI and HOMA-IR	Decrease in BW	Amelioration of lipid profile
Leite et al., 2022 [161]	Aerobic	Exe and Con: 13.00 ± 1.90	12	Nutritional guidance in Con, Habitual diet in Ex	Decrease in FI, FBG, HOMA-IR and QUICKI	Decrease in BW and BMI	Amelioration of lipid profile
Salahshoornezhad et al., 2022 [162]	Aerobic	Exe and Con: 10.50 ± 1.02	10	na	Decrease in FBG	Decrease in BW and WC	Amelioration of lipid profile
Alizadeh et al., 2019 [163]	Aerobic	Exe and Con: 18.00 ± 1.5	6	Habitual diet	Decrease in FBG FI and HOMA-IR	Decrease in BW	na
Kim et al., 2020 [147]	Aerobic	Exe and Con 15.00 ± 1.00	12	Habitual diet	Decrease in FBG FI and HOMA-IR	Decrease in BW	Decrease in blood pressure levels
Murphy et al., 2009 [148]	Aerobic	Exe and Con: 10.21 ± 1.67	12	Habitual diet	na	Decrease in BW	Decrease in blood pressure levels and lipid profile
Vasconcellos et al., 2015 [149]	Aerobic	Exe1: 16.60 ± 0.90 Exe2: 16.50 ± 1.20 Con: 16.90 ± 1.00	12	Habitual diet	Decrease in HOMA-IR	Decrease in BW and WC	Decrease in fat mass, blood pressure, and amelioration of lipid levels
Boer et al., 2014 [164]	Aerobic	Exe1: 18.00 ± 3.20 Exe2: 16.70 ± 3.60 Con: 17.40 ± 2.40	15	Habitual diet	Decrease in FBG, FI, and HOMA-IR	Decrease in BW	Decrease in body fat, blood pressure levels and amelioration of lipid profile
Resistance training							
Benson et al., 2008[165]	Resistance	Exe and Con: 12.20 ± 1.3	8	Habitual diet	Decrease in FBG, FI	Decrease in WC	Decrease in body fat
Kelly et al., 2019 [166]	Resistance	Exe: 15.29 ± 0.95 Con: 15.58 ± 0.99	16	Habitual diet	Decrease in FBG	na	na
Lee et al., 2013 [167]	Aerobic vs. Resistance	Exe1: 14.60 ± 1.90 Exe2: 14.80 ± 1.90 Con: 15.00 ± 2.20	12	Habitual diet	Decrease in FI and HOMA-IR	na	Decrease in visceral fat
Lee et al., 2010 [168]	Aerobic vs. Resistance	Exe1: 15.20 ± 1.90 Exe2: 14.60 ± 1.50 Con: 14.80 ± 1.40	12	Habitual diet	Decrease in FI	Decrease in BW	na
Rasooli et al., 2021 [169]	Resistance	Exe and Con: 14.00–17.00	8	Habitual diet	Decrease in FI and FBG	Decrease in BW	na
Seo et al., 2012 [150]	Resistance	Exe: 14.70 ± 1.51 Con: 14.60 ± 3.03	8	Balanced diet	Amelioration of IR	Decrease in BW	Amelioration of lipid profile
Combined training							
Chae et al., 2010 [170]	Combined (Aerobic and Resistance)	Exe: 10.60 ± 3.80 Con: 10.40 ± 3.10	12	<1800–2000 kcal/day	na	Decrease in BW	Improvement of body composition and serum lipid profiles
Vissers et al., 2008 [171]	Combined (Aerobic and Resista ce)	Exe (F): 17.50 ± 1.30 Exe (M): 18.10 ± 1.30 Con (F): 17.10 ± 1.10 Con (M): 17.50 ± 1.40	24	Counseling by a dietitian	Decrease in FBG	Decrease in BW and WC	na
Wong et al., 2018 [172]	Combined (Aerobic and Resistance)	Exe: 15.20 ± 1.20 Con: 15.30 ± 1.10	12	Balanced diet	Decrease in FI and FBG	Decrease in BW	Decrease in body fat
Sefat et al., 2019 [173]	Combined (Aerobic and Resistance)	Exe: 12.40 ± 1.71 Con: 11.80 ± 2.20	8	Habitual diet	Decrease in FI, FBG, and HOMA-IR	Decrease in BW	na
Son et al., 2017 [174]	Combined (Aerobic and Resistance)	Exe and Con: 15.00 ± 4.47	12	Habitual diet	Decrease in FI, FBG, and HOMA-IR	Decrease in BW and WC	na
Zehsaz et al., 2016 [175]	Combined (Aerobic and Resistance)	Exe: 10.80 ± 0.90 Con: 10.30 ± 0.90	16	Habitual diet	Decrease in FI and HOMA-IR	Decrease in BW and WC	Decrease in body fat and amelioration of lipid profiles
Davis et al., 2011 [176]	Combined (Aerobic and Resistance)	Exe: 15.70 ± 1.10 Con: 15.80 ± 1.00	16	Habitual diet	Decrease in FI and HOMA-IR	Decrease in WC	Decrease in body fat
Farpour-Lambert et al., 2009 [177]	Combined (Aerobic and Resistance)	Exe: 9.10 ± 1.40 Con 1: 8.80 ± 1.60 Con 2: 8.50 ± 1.50	12	Habitual diet	na	Decrease in BW	Decrease in blood pressure levels
Lopes et al., 2016 [178]	Combined (Aerobic and Resistance)	Exe: 14.60 ± 1.15 Con: 14.40 ± 1.16	12	Habitual diet	Decrease in FBG, FI, and HOMA-IR	Decrease in BW	na
Davis et al., 2009 [179]	Resistance Combined (Aerobic and Resistance)	Exe1: 15.70 ± 1.20 Exe2: 14.80 ± 1.00 Con: 15.3 ±0 1.10	16	Nutritional education class	No significant changes in FBG and FI	Decrease in BW	Decrease in body fat
de Lira et al., 2017 [180]	HIIT LIT	Exe1: 14.95 ± 1.35 Exe2: 14.77 ± 0.94 Con: 14.72 ± 1.35	12	Balanced diet	No significant changes in FBG FI HOMA-IR	na	Improvement of biomarkers related to non-alcoholic fatty liver disease
Meng et al., 2022 [181]	HIIT MICT	Exe1: 11.4 ± 0.80 Exe2: 11.2 ± 0.70 Con: 11.0 ± 0.70	12	Habitual diet	Decrease in FI and HOMA-IR	Decrease in BMI	na
Plavsic et al., 2020 [182]	HIIT	Exe: 16.20 ± 1.30 Con: 15.50 ± 1.50	12	1500–1700 kcal/day	No significant changes in metabolic parameters	Decrease in BW	na
Abassi et al., 2020 [183]	HIIT MIIT	Exe1: 16.10 ± 0.99 Exe2: 16.50 ± 1.07 Con: 16.90 ± 1.64	12	Habitual diet	Decrease in FI and HOMA-IR	Decrease in BW	Decrease in body fat
Racil et al., 2016 [184]	HIIT Combined (Plyometric exercise and HIIT)	Exe1: 16.60 ± 0.90 Exe2: 16.50 ± 1.20 Con: 16.90 ± 1.00	12	Habitual diet	Decrease in FBG, FI, and HOMA-IR	Decrease in BW	Decrease in blood pressure levels
Dias et al., 2018 [185]	HIIT MICT	Exe1: 12.40 ± 1.90 Exe2: 11.90 ± 2.40 Con: 11.8 ± 2.40	12	Balanced diet	No significant reductions in metabolic parameters	na	No significant reductions in metabolic parameters
Racil et al., 2013 [186]	HIIT MIIT	Exe1: 15.60 ± 0.70 Exe2: 16.30 ± 0.52 Con: 15.90 ± 1.20	12	Habitual diet	Decrease in FI and HOMA-IR	Decrease in BW	Amelioration of lipid profile
Meyer et al., 2006 [187]	Swimming and Aqua aerobic training + Sports games + Walking (combined)	Exe and Con: 14.70 ± 2.20	24	Habitual diet	Decrease in FI and HOMA-IR	na	Decrease in body fat, blood pressure levels, CV risk, and amelioration of lipid profile
Others							
De Souza et al., 2022 [188]	Karate training	Exe and Con: 12.00–17.00	12	Nutritional plan	Decrease in FBG	na	Amelioration of lipid profile and heart rate

WC: waist circumference; Con; control; Exe: exercise; FBG: fasting blood glucose, FI: fasting insulin, HOMA-IR: Homeostasis model assessment: insulin resistance, BW: body weight, IR: insulin resistance, Exe: exercise group, Con: controls, HIIT: High-Intensity Interval Training, MICT: moderate-intensity continuous training, MIIT: moderate-intensity interval training, LIIT: Low-Intensity Interval Training, na: not available; CV: cardiovascular; QUICKI: quantitative insulin sensitivity check index.

## 5. Limits

We recognize that this review has some limitations. Firstly, we present a non-systematic overview and analysis of the existing literature on a particular topic. The non-systematic nature of narrative reviews [18] implies that there are no formally established guidelines for their conduct, potentially introducing selection biases of the literature and often leading to qualitative rather than quantitative syntheses. For instance, our review only considered articles available on PubMed and Scopus, meaning that relevant studies indexed in other databases or search engines might have been inadvertently excluded.

Secondly, in most studies describing the impact of physical exercise on glucose metabolism and IR in the pediatric age, the variations of myokines during training or after a specific exercise time program are not reported. Therefore, it is difficult to qualitatively and quantitatively assess their different roles in various types of activities and to drive more specific conclusions about their particular role in this context.

Additionally, longitudinal studies on long-term follow-up are limited. Further studies are necessary to better evaluate the differences among exercise modalities in terms of outcomes and long-term follow-up.

Finally, the reported studies do not examine in detail the effects of exercise during the transition from childhood to adolescence and from adolescence to adulthood, periods in which both muscle structure and metabolism undergo significant physiological changes. Comprehensive and multidisciplinary studies are necessary to fully understand the pathogenetic mechanisms underlying the association between skeletal muscle health and metabolic balance during growth.

## 6. Conclusions

Skeletal muscle is recognized as an endocrine organ. The analysis of various molecules produced and secreted by skeletal muscle reveals that the skeletal muscle secretome, through its paracrine and endocrine functions, significantly contributes to the maintenance and regulation of overall physiological health. Specifically, myokines such as IL-6, IL-8, and IL-15, as well as irisin, myonectin, and myostatin, appear to play a crucial role in IR. 

Unfortunately, evidences concerning the role of other exercise-induced molecules in glucidic metabolism in the pediatric field are still limited thus, other studies are needed. Additionally, in children with obesity, skeletal muscle can also become a target of obesity-induced and IR-induced inflammation. In the correlation between muscle, IR, and inflammation, the role of infiltration by immune cells and the microvasculature as the site of insulin action and delivery may also be considered.

In the pediatric population, exercise training, regardless of the type of exercise chosen, was shown to have beneficial effects on glycemic metabolism—evaluated with FBG, FI, and HOMA-IR—as well as on BW. This is extremely important to prevent IR, T2D, and the deleterious consequences of these conditions during childhood and adolescence. Physical activity should be recommended as adjunctive therapy to control IR and related outcomes in these patients. It is still unknown whether the effect of exercise training would be similar in both biological sexes, across different ranges of ages (young children vs. adolescents), and whether they would be dependent on overweight or obesity status or other health conditions. Thus, further investigations should focus on these factors to determine the best recommendations. 

In addition, it remains unclear which exercise approach is the best; however, aerobic exercise is highly effective in improving insulin sensitivity in children and adolescents with obesity. However, when combined with RT, specifically with HIIT, the benefits seem to be amplified, making this combination the most effective strategy for managing IR. High-intensity activities offer superior metabolic advantages and are often more enjoyable for children, which increases the likelihood of long-term adherence. Therefore, it is crucial to encourage daily participation in these activities, with a strong emphasis on making exercise fun and engaging to ensure consistent, long-term commitment and effective management of glucose metabolism. 

Even though the choice of sport should be made considering the child’s physical and cognitive abilities, as well as the coordination skills that can be developed at different ages, at every age, physical activity can be recommended. It is essential to educate people from childhood to develop the habit of engaging in daily physical activity so that it becomes a part of their lifestyle as they grow older. Through education in movement, the developmental process is enhanced and completed, contributing to achieving balanced growth and development and skeletal muscle health while preventing diseases in later stages of life.

The development of strategies and actions to promote physical activity in children and adolescents must be a top priority for public health, not only in terms of individual quality of life and well-being but also for the health of the community, contributing to the reduction in direct and indirect healthcare costs. 

## Figures and Tables

**Table 1:** Myokines and its main functions.

Myokines	Function
Myostatin [22,27,29,30,31,32,33]	It increases with physical inactivity. It is an inhibitor of muscle mass gain and bone healing. It is involved in metabolic equilibrium and the control of adipose tissue activity and mass. It is regulated by decorin and follistatin.
IL-6 [4,7,23,28,36,37,39,40,41]	It is involved in autocrine and paracrine signaling in skeletal muscle, particularly activating the STAT3 pathway, which is important for hypertrophic muscle growth and myogenesis. It enhances insulin-stimulated glucose uptake and oxidation, stimulates lipolysis and fat oxidation, promotes pancreatic β-cell expansion, and improves insulin secretion and glycemic control. IL-6 produced by skeletal muscles during exercise exerts anti-inflammatory effects, suppressing the synthesis of IL-1 and TNF-α and stimulating the production of anti-inflammatory cytokines like IL-1ra and IL-10.
IL-15 [4,28]	It is an anabolic factor that stimulates muscle growth: IL-15 signaling is involved in the regulation of muscle fiber composition and contractility. It is involved in lipid metabolism regulation, reducing lipid accumulation in preadipocytes and decreasing the mass of white adipose tissue. A negative relationship has been observed between plasma IL-15 levels and total body fat mass, especially trunk fat mass.
Decorin [33,43,46]	It plays a role in the regulation of muscle hypertrophy, particularly in response to exercise. Together with follistatin, it is a myostatin inhibitor.
Leukemia inhibitory factor (LIF) [22,44,47,48]	It is a contraction-induced myokine. It acts in an autocrine and/or paracrine fashion. It promotes platelet production, the proliferation of hematopoietic cells, bone formation, neural survival and development, and the acute-phase response in liver cells. It supports satellite cell proliferation.
Chitinase-3-like protein 1 (CHI3L1) [45,49,50]	It is produced and secreted by skeletal muscle in response to acute physical exercise. Through the CHI3L1/PAR-2 signaling pathway, it stimulates myocyte proliferation, which is important for skeletal muscle remodeling in response to training. It is linked to increased glucose uptake in skeletal muscles both via an AMP-activated protein kinase (AMPK)-dependent mechanism and via the PI3K/AKT pathway.
Angiopoietin-like 4 (ANGPTL4) [51,52,53,54,55,56]	It inhibits lipoprotein lipase activity, which reduces lipid accumulation and promotes lipolysis in white adipose tissue. In non-exercising muscles, ANGPTL4 levels increase in response to elevated plasma FFA through PPAR activation, helping to prevent fat overload and ensuring the supply of fatty acids to active skeletal muscles. By regulating its expression through PPARs, ANGPTL4 may contribute to the development of insulin resistance. ANGPTL4 levels can be modified by exercise, such as acute resistance and strength training, as well as by dietary factors, particularly the intake of SFA. It has been implicated in mice as a mediator of pancreatic β-cell hyperplasia.
Apelin [57,58,59,60,61]	It enhances glucose uptake in skeletal muscle. It increases mitochondrial oxidative capacity in skeletal muscle. It improves muscle function by enhancing energy production. It reduces inflammation in skeletal muscle. It supports muscle stem cell (MuSC) regeneration, especially stimulating skeletal muscle endothelial cells (ECs).
Irisin [22,62,63,64]	It is a polypeptide hormone produced by muscles in response to physical exercise. It promotes the conversion of white adipose tissue into a brown fat-like phenotype. It exerts antifibrotic effects on the heart, liver, pancreas, and muscle.
VEGF [65,66]	It is a primary angiogenic factor that promotes the growth of new blood vessels, including the capillaries within skeletal muscle. During physical exercise, VEGF levels rise in the muscle interstitium, enhancing angiogenic activity. It facilitates the development and growth of blood vessels within skeletal muscle tissue, ensuring adequate blood supply to the muscles. It contributes to the regeneration of tissues, supporting repair and recovery processes in skeletal muscle.
IL-8 [22]	It induces angiogenesis.
CYR61 (CCN1) and CTGF (CCN2) [67]	They increase after physical activity, especially following mechanical loading. They regulate the expression of genes involved in angiogenesis and ECM remodeling.
Brain-derived neurotrophic factor (BDNF) [28,68,69]	Its levels increase after physical activity. It plays a critical role in the growth, survival, and maintenance of neurons. It impacts information processing, learning, and memory. It influences energy metabolism by enhancing fat oxidation in skeletal muscles through an AMPK-dependent pathway. It contributes to a decrease in adipose tissue mass.

## Data Availability

Not applicable.
